# A conversation on using chemical probes to study protein function in cells and organisms

**DOI:** 10.1038/s41467-022-31271-x

**Published:** 2022-07-05

## Abstract

Chemical probes are selective small-molecule modulators, usually inhibitors, of their target protein’s function, that can be used in cell or even animal studies to interrogate the functions of their target proteins. Cheryl Arrowsmith, the leader of a new initiative called Target 2035, which seeks to identify a pharmacological modulator for most human proteins by the year 2035, and Paul Workman, the Executive Director of the nonprofit Chemical Probes Portal, an online resource dedicated to chemical probes, talked to *Nature Communications* about chemical probes, their respective paths to leadership positions in the field, the online resources available to those interested in the topic and the promise and value of open — collaborative — science. The below material is a modified transcript of a long discussion, preserving the conversational tone, but streamlined and edited for clarity, and thus we do not attribute the particular parts to Cheryl or Paul specifically except for when they shared their personal experiences.

Cheryl Arrowsmith (ORCID: 0000-0002-4971-3250) is the Chief Scientist for the Structural Genomics Consortium (SGC) Toronto laboratories, Professor of Medical Biophysics at the University of Toronto, and Senior Scientist at the Princess Margaret Cancer Centre. She is a Fellow of the Royal Society of Canada and Fellow of the American Association for the Advancement of Science. She is leading a new initiative called Target 2035, which seeks to identify a pharmacological modulator for most human proteins by the year 2035 (www.Target2035.net). Her personal research interests focus on understanding and exploiting epigenetic mechanisms in cancer using chemical biology approaches, especially development and application of chemical probes.

**Figure:**
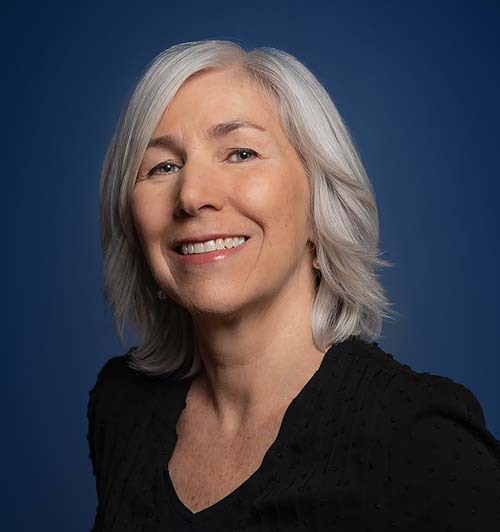
University of Toronto

Paul Workman (ORCID: 0000-0003-1659-3034) is Harrap Professor of Pharmacology and Therapeutics at the Institute of Cancer Research (ICR) in London, having served until recently as the institution’s President and Chief Executive and Director of the ICR’s Cancer Research UK Cancer Therapeutics Unit. He is a Fellow of the Royal Society (the UK’s national academy of science), Fellow of the Academy of Medical Sciences, the Royal Society of Chemistry and the Royal Society of Biology, and the European Academy of Cancer Sciences, and is a Cancer Research UK Life Fellow. Paul currently serves as the Executive Director of the nonprofit Chemical Probes Portal (www.chemicalprobes.org), an online resource dedicated to chemical probes. In his personal research, he has been instrumental in the discovery of numerous chemical probes and cancer drugs.

**Figure:**
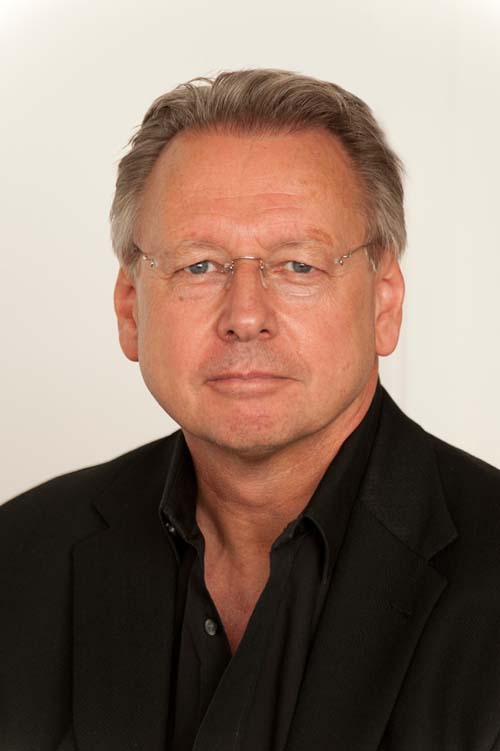
Jan Chlebik/Institute of Cancer Research, London

1. What are chemical probes?

— A chemical probe should be a potent and selective ligand of its target protein that modulates the biochemical activity of the target in cells. Ideally, it should have well-characterized selectivity, especially with respect to closely related proteins. It should be active in cells at a reasonable concentration, typically in the low micromolar or nanomolar range, and it cannot be generally toxic. With those properties, one can confidently use a molecule as a probe of the target protein function in a cellular context. If the molecule has good pharmacological properties—good pharmacodynamics and pharmacokinetics—then it can be also used in a model organism.

— Chemical probes most commonly act as inhibitors but they could be activators, too. Biomedical researchers use chemical probes to determine their respective target’s role in biological processes or disease pathology. And scientists can also use such high-quality chemical probes to help validate drug targets, alongside the application of orthogonal genetic methods. It’s worth mentioning that the term ‘chemical probe’ is used in other contexts for lab reagents that are, for example, labelled for use in biophysical or imaging studies. However, those are not what we’re talking about here. Again, chemical probes are small-molecule modulators to interrogate the functions of their target proteins, as opposed to protein location, or other physical properties. One of our goals is that researchers across different disciplines understand the name “chemical probe” this way and use this terminology consistently.

2. What made you personally interested in chemical probes?

**Paul Workman**

I trained in biochemistry and pharmacology, so when I came to develop my career in cancer research and drug discovery I was already accustomed to using small-molecule inhibitors, particularly enzyme inhibitors, to perturb the activity of proteins and understand their function. It was kind of obvious and second nature to me. But there were two particular experiences that further crystallized my zeal.

The first was in my pharma industry period between 1993 and 97 where I led much of the work to define and exploit new protein targets for cancer drug discovery in what is now AstraZeneca. There, working with medicinal chemists and pharmacologists, I learned the importance of what in industry is commonly termed a ‘tool compound’. A tool compound doesn’t necessarily need to achieve the very top level of stringent criteria as the best chemical probes, but it certainly needs to be of good enough quality to help the research team determine if they’re on the right track: for example, to see if inhibiting the protein target’s activity results in blocking a cancer cell growth pathway. This is a huge help with target validation and can point the way toward a future drug.

The second influential experience was during the same period, when I joined the consortium of pharmaceutical companies working on kinase inhibitors with Philip Cohen’s team at Dundee University. In the year 2000, Philip’s group published, for the first time, a limited but quite rigorous and systematic assessment of kinase inhibitor specificity, showing how well a particular kinase inhibitor blocks its intended target and at the same time also assessing if it inhibited any other kinases—which they frequently did^1^. This was the origin of kinase selectivity testing panels which were not standard at the time. The results were often very surprising—with some compounds inhibiting multiple kinases, including ones not closely related structurally or functionally to the main putative target. Working with this team, and a small group of companies, really exemplified the value of more detailed characterization of chemical probes—and also drugs and drug leads. This allowed kinase researchers to rigorously select the best chemical probes, discarding inferior ones. Our experience also showed the value of using more than one probe to improve the experimental robustness of findings by reducing the risk of off-target effects.

Those were real wake-up moments, when I became even more aware of the power of high-quality chemical probes, but also the dangers associated with using bad reagents, or even just suboptimal ones. The ‘promises and perils’ as we wrote later^2^.

**Cheryl Arrowsmith**

I came into the field a little bit differently. I was trained as a chemist, but for most of my research career I have been a structural biologist, thinking about proteins, how they function and how to modulate them.

Chemical probes were really highlighted to me in conversations with Timothy Willson, who in the 1990s led GSK’s program to use chemistry to illuminate the biology of nuclear hormone receptors. His work started before the receptors were all cloned and characterized. The estrogen receptor, the androgen receptor, these were known, but there’s a whole family of them. Tim and his team identified these uncharacterized (orphan) nuclear receptors (NR) through sequence homology and cloned them. But what they also did, that was innovative at the time, was systematically identify ligands that would modulate the functions of these proteins. They developed very potent and selective small molecule chemical probes (inhibitors or agonists), but, in order to fully explore the biology of these proteins, they really needed to work with experts in biology of diseases—outside the company. They did that through individual material transfer agreements with each institution; it was a case-by-case, time-consuming process, but it ultimately led to the discovery of new NR-mediated biology and several successful drug programs. By the 2000s, Tim had made many of the compounds available through vendors such as Sigma, but told me, in retrospect he felt that if they had been able to systematically make their chemical probes available to the entire research community earlier, we probably would have understood the NR family more quickly.

The data seems to support this notion. There’s this great plot showing the number of publications on the individual members of the NR family and the availability of chemical probes for these proteins^3^. The biology is best understood for those family members that have selective inhibitors to them, and the rest of the family members remain less well studied and understood to date. At the SGC we have been systematically developing open source chemical probes for several protein families and providing them to the community to accelerate our understanding of each protein and of human biology and disease.

And, as Paul points out, these compounds have to be good quality molecules for that purpose, and scientists using them need to know how to use them properly, under the right circumstances. So we do spend a lot of effort to both make high-quality probes, and also to provide all the key data that will enable biologists to use them appropriately.

**Paul Workman**

That period, around the late 1990s and early 2000s, was really a critical time for the field. The developments in the kinase and the nuclear hormone receptor areas, also now with epigenetic modifiers, are really great examples of the systematization of chemical probe development, and the value of open source research—with contributions from both academia and biopharmaceutical companies, often working successfully in partnership. And of course it was also the time when the human genome sequence came online and opened up a superb wealth of opportunity. But this resulted in a new bottleneck which was to understand protein function in basic research and validate drug targets, both on a much bigger scale—which chemical probes have proved super crucial for.

What also happened during that time period, which had both mostly beneficial and initially some adverse consequences, was an explosion of interest in academic drug discovery and chemical biology that led to many academic labs running large-scale chemical compound screens—often using phenotypic cell-based assays—in search of potential drugs or chemical probes. This resulted in many publications that often concluded that the researchers had discovered a chemical probe—but what in fact they’d done was to identify an initial hit compound. Many of these compounds were not very well characterized—they commonly weren’t very potent or selective toward the proposed target protein—and some were of very poor quality, acting non-selectively and sometimes very promiscuously—at worst behaving as ‘chemical con artists’^4^. We continue to see such compounds touted as probes and drug leads, for example in Covid-19 screening. Using such poor-quality compounds in biology experiments gives misleading or completely wrong information about the role of the intended target. It takes a lot of work to make a high-quality chemical probe. They don’t just fall directly out of screens. The screening hit is a starting point. It typically requires several chemists and several biologists to work together for two or three years to optimize the structure of the hit into a high-quality, well-characterized probe. Unfortunately, in those early days, there commonly wasn’t the resource or expertise in academia to follow up the output from initial screens, to profile the hits, and make the compounds more potent and selective. The widespread use of compounds that are insufficiently characterized and promiscuously active—or just not sufficiently selective or simply out of date, continues to be a major problem across biomedical research. It wastes researcher’s time and resources, leads to incorrect conclusions about protein function and target validation, and even results in worthless compounds progressing to clinical trials.

But at the same time, the growth of the chemical biology field in academia had a really positive side. Previously the knowledge about what makes a high-quality chemical probe—and what the potential problems and dangers are—largely resided in the biopharmaceutical industry. For example, medicinal chemists working in industry had the experience to recognize the potential for a compound to have serious problems like being chemically reactive or redox active, a colloidal aggregator or toxic. They had seen multiple examples of ‘frequent hitters’. This knowledge wasn’t so common in academia at the time, but it has since been developed strongly in chemical biology labs. However, we still have a lot of work to do to deal with the problem of the misuse of problematic ‘nuisance’ compounds as probes in the biomedical research community and to promote the development and best practice use of high-quality chemical probes.

3. What are the most important considerations for developing a good chemical probe?

— The first essential thing that needs to be done is to eliminate the really bad nuisance compounds, which can have problematic behavior—like being non-specifically very reactive with proteins; forming colloidal aggregates that non-specifically adsorb and inactivate proteins; exerting toxicity toward cells, for example through a membrane damaging effect called phospholipidosis; or exhibiting spectral or fluorescence properties that interfere with the biological assay read-out. These undesirable compounds are often referred to as Pan Assay Interference or PAINS compounds, as highlighted by Jonathan Baell^4^. There are software filters or algorithms available that should be used routinely to identify any risk of such chemical promiscuity and simple lab assays should be run to check for the various problematic properties we mentioned. Such compounds should never be considered further or used as chemical probes. They should be excluded from compound libraries. Yet many are sold by commercial vendors as chemical probes and widely used.

Beyond kicking out the most egregiously horrible nuisance compounds, the selection of the best possible quality chemical probe is greatly helped by applying rigorous criteria. These include the ‘fitness factors’^5^ and the guidelines and related criteria developed by the SGC and a team of international experts from academia and industry^2^. High potency against the target is essential, with IC_50_ or K_d_ values of 30–100 nM or lower on the desired target. Activity in cells via the intended mechanism should be seen at concentrations no higher than 1–5 μm or at most 10 μm and, ideally, sub-micromolar. The more potent the compound is, the less likely it is you will have off-target effects.

— What you also need is very detailed characterization, testing especially the compound for selectivity against as many proteins as possible. Selectivity is super important. We think a good probe should be at least 30-fold more selective compared to proteins in the same family. Broader profiling and against representative pharmacology panels, as used in industry for off-target mitigation and safety prediction of lead compounds and drug candidates, is also very valuable. These panels—including ion channels, G protein-coupled receptors, neurotransmitter transporters, enzymes, and nuclear hormone receptors are offered by contract research organizations and a consensus minimum panel of around fifty targets has been defined. Such broad profiling is especially useful for chemical probes to be used in animals. A high-quality chemical probe should hit only a small number of off-targets and it’s important that these data are made available, so that results can be interpreted accordingly.

By the way, this is where the use of open science, of sharing the compound with the entire scientific community, is enormously useful. If you put a high-quality probe compound out into the community, researchers will rigorously characterize and determine the value of the compound. This will include evaluation in a wide range of cellular systems and model organisms.

— Obtaining evidence of target engagement and target inhibition in cells is also critical. Many different screens can now be applied to compounds to see whether they’re broadly reactive, or promiscuously modulatory, or at least whether they bind to many proteins in the cell or not. These screens are really necessary. You commonly see publications reporting that a proposed probe, which perhaps shows promising activity on the isolated target in vitro, is then tested in cells and elicits the anticipated response—often the killing of cancer cells—leading to the conclusion of cause and effect. But that effect could be non-specific, unrelated to the intended target. Demonstrating binding and modulation of target activity inside living cells, and also ideally that downstream pathway modulation actually does occur inside the cells—at a sensible concentration that relates to the corresponding in vitro activity—is really crucial. Being able to mechanistically link up the effects of a probe in a solid Pharmacological Audit Trail is very important^6^.

— Another key point is that best practice covers not only the selection of the highest quality available chemical probes but also the critical application of these^7^. This entails using, if available, two different chemical probes with dissimilar chemical structures (known as chemotypes) and also ideally the corresponding inactive control compounds—that is related chemical analogs that are inactive or very, very much less active than the probe itself on the target of interest. Using two chemically unrelated active probes for the same target gives greater confidence in the robustness of the results, because the two chemotypes should ideally not have the same off-targets. So any observed phenotypic effects seen with both of the probes are more likely to be a consequence of engaging their common target than is the case with a single probe—giving more robustness compared to only a single probe being used. However, you have to be careful in choosing your inactive controls. A chemical substitution that takes out the activity against the on-target, may also remove the off-target activities, and that can muddle the interpretation. So you need to profile the inactive controls as well and interpret the findings in the light of all of the data^8^.

A recent paper by Jason Sheltzer’s group highlighted that it can be valuable to look at the phenotypic consequences for a small-molecule inhibitor of removing the target protein target completely from cell^9^. If a drug or chemical probe retains the phenotypic effect in cell clones in which the putative target is knocked out by CRISPR, this must mean that the phenotype is not mediated by that target but rather involves off-target activity. This approach has been used to show very clearly that several cancer drugs already in clinical trials do not actually act through their assumed targets—because they retain anticancer activity in cells that lack the respective target proteins. If the phenotypic activity in the CRISPR knockout clones is lost compared to wild type cells this indicates that inhibiting the proposed target is important for the phenotype. Another useful test that can be used as a genetic gold standard is whether, as expected, the phenotype of the drug or probe is lost in cells expressing a mutated form of the proposed target that confers resistance to the targeted inhibitors.

Much of this is about having a critical mindset, using rigorous controls and avoiding confirmation bias. This is true both for chemical probe developers and equally for chemical probe users. If one has produced a candidate chemical probe, it’s really super important to be very critical, deploying a range of experiments that might disprove its validity as a chemical probe for a given target, or highlight limitations that can be factored into the use of the probe. And if one is looking to choose and use a chemical probe then be super careful to understand it’s strengths and limitations. Remember ‘caveat emptor’ – ‘Let the buyer beware’.

— If meeting the recommended standards for a high-quality chemical probe seems like a huge effort, we need to consider the enormous cost of not doing this well. We have estimated that the cost of the academic research done on poor quality nuisance compounds runs into billions of dollars, and the costs are even higher if a compound progresses to be tested as a drug. A very cautionary example is the case of iniparib—referred to as ‘the PARP inhibitor that never was’^10^. It actually does not behave as a bona fide inhibitor of PARP [poly(ADP-ribose) polymerase]. Medicinal chemists will immediately recognize several fatal flaws in the compound structure, including likely instability and chemical reactivity. Indeed, proteomic analysis revealed that it reacts promiscuously with cysteines in large numbers of cellular proteins. Despite apparently limited and uncritical characterization, iniparib progressed into a Phase 3 clinical trial where it failed to show activity in breast cancer. Over 2500 patients were treated. The costs would have been enormous—likely hundreds of millions of dollars. And the negative results endangered the entire PARP field for a period. Inexcusably, iniparib is still sold by commercial vendors as a PARP inhibitor. Fortunately, we now have several high-quality PARP inhibitors that are approved for use in cancer patients and are very valuable chemical probes.

4. Chemical probes can also be used in model organisms. What are the most important considerations for such use?

— Even after the compound is successfully validated as a bona fide chemical probe to use in cell culture or in zebrafish embryos or similar simple systems, a natural next step is often to use the probe in a model animal, most often a rodent. But in the literature, it’s very common to see the proposed agent administered at a dose for which there is no justification provided and often no pharmacokinetic data reported^11^. So there is frequently no measurement of blood levels, to know whether the probe is circulating at concentrations that are needed to modulate the target—let alone measurements of its tissue levels, to tell you that the compound gets to where its intended target is located. So how can we know if the compound has achieved the appropriate level of exposure that would support modulation of the desired protein target for the necessary duration? There’s also a further refinement involving the concept of the free drug concentration—which is the amount of drug that’s not bound to proteins in the plasma and so is available to act on the intended target. This is frequently not measured or at least not reported either. As a researcher you need to know the level of the chemical probe in the plasma and the tissue. If you don’t have that, you can’t interpret what the results mean, you can’t firmly link the phenotype in the animal to on-target modulation. Essential criteria for chemical probes in mice, as advised by information on the Chemical Probes Portal, are the elimination half-life, systemic clearance, fraction of the compound that is protein-bound, maximum plasma concentration after drug administration and time to reach maximum plasma concentration for the given dose. And additional criteria apply for chemical probes that need to enter the brain.

In addition to pharmacokinetics, ideally pharmacodynamic data would be obtained, involving measuring biomarkers that inform on the degree of target modulation—the Pharmacological Audit Trail again^6^.

5. What then is the difference between a chemical probe and a drug?

— Historically, although crucial contributions were made with the advent of chemical biology, it has mainly been industry that had the resources to do the very multi-disciplinary and, frankly, expensive work to make a good quality chemical tool. Pharmaceutical companies did this with the aim of developing a drug, intended to treat disease. And until fairly recently, the biologists in academia mostly only had drugs (or analogs of them) to use as small-molecule modulators in their experiments. If an inhibitor was made by a pharmaceutical company, the biologists commonly just trusted it without really thinking about the details. But drugs are different from chemical probes. Drugs don’t necessarily need to be as selective as high-quality chemical probes. They just need to get the job done on the disease and be safe to use. In fact, many drugs act on multiple targets as part of their therapeutic mechanism. Researchers need to consider the chemical tools that they are using, and if they want to use a drug, they need to validate it first as a bona fide chemical probe. Biologists are used to validating their reagents: shRNAs or antibodies, etc, and they know the importance of selectivity and controls. They also need to think hard about off-targets and controls for chemical probes, and they need to validate both probes and inactive control compounds, or at least find the validation data in the literature, and cite the relevant papers in their own work. The same applies to compounds that are commercially available as claimed probes. Vendor catalogs often describe compounds as chemical probes or inhibitors of a particular target, but the actual evidence for these claims is often missing on their websites. This is where the Chemical Probes Portal is so useful.

6. You’ve mentioned the Chemical Probes Portal and the website of the Target 2035 initiative, which are both great resources dedicated to chemical probes. Could you introduce them to our readers?

—The Chemical Probes Portal came about because of all the potential benefits and challenges that we’ve just been talking about. A high-quality chemical probe is really powerful, complementary to orthogonal genetic methodologies—really super valuable. But it has to be high quality. If it’s not high quality, its effects might be reproducible—one can reproducibly repeat a meaningless experiment—but the findings are not robust to the question that’s being asked about the function of the protein target, like its role in biology and disease and as a target for drug treatment. So what is needed for the biomedical research to get the best out of chemical probes?

Firstly, it’s obvious that we need many, many more high-quality chemical probes to functionally annotate the whole proteome and identify more new drug targets at scale. And secondly, we to need to develop a better understanding across the broad biomedical research community of what makes a high-quality chemical probe and how to use them in a best practice fashion—while also eliminating the use of the very poor quality and frankly dreadful reagents. The first is a science and logistics challenge. The second is a training and communication challenge.

In terms of proteome coverage, our calculation is that <10% of proteins in the proteome have even a minimal criteria chemical probe, which leaves us with 90% of proteins—even some very well-known ones, let alone the ‘dark proteome’—that don’t have a targeting small molecule^12^. The Target 2035 initiative is addressing the need to get more probes: more targets covered and more protein families covered. The Chemical Probes Portal was set up to ensure that researchers have the information to choose the right chemical probes and use them well.

Ultimately, chemical probes are used by biologists to ask biological questions. But the expertise needed to choose the right probe is just not in the training of most biologists. Also, the information on chemical probes is complex and can be very widely distributed. So where can you find it? Typically, researchers look to find a chemical probe for their target using Google- or PubMed-type searches. Often, by looking for the most cited paper or reagent. Unfortunately, that approach will often suggest a very early compound, which might have been useful 20 years ago and is therefore highly cited, but might have been discredited or superseded by now. People also look in the commercial vendor catalogs, but these are not always the best source of information either.

Given the wealth of data available, and the challenge of finding a way through this—especially for non-experts—online public resources on chemical probes can be super-useful to researchers^12^. When we launched the Chemical Probes Portal in 2015^2^, we envisaged this as a user-friendly go-to resource, where researchers could find expert advice and reliable information about chemical probes. It’s a website, you type in the name of the target that you’re interested in, and it will bring up the information you need: probes that have been reviewed by members of the Portal’s large Scientific Expert Review Panel. The probes are scored and reviewers’ specific comments are also provided. For example, the advice may suggest that more extensive selectivity profiling of a probe is needed. Or that you should use it over a particular concentration range. Or that the probe is excellent for use in cell models, but is not validated yet for use in animals, because there’s no pharmacokinetic data. Or the review may indicate that the probe has got one or more off-targets that might be confounding, so one really must use a second probe alongside it that doesn’t inhibit that particular off-target, as we mentioned earlier. At the very least, the reviews will make you aware of the benefits and the limitations. And you will be also signposted to further information and publications if you need to dig deeper. The Portal features, as well, a variety of information that can be particularly useful to non-specialists.

— Currently, the Portal has 739 compounds, close to 500 of those are really good high-scoring quality chemical probes. We also have information on what we refer to as ‘historical compounds’—these include problematic compounds and earlier tools that have been superceded and no longer recommended. To date, 380 protein targets are covered. There’s nearly a thousand reviews.

It’s worth mentioning that the expert review basis of the Portal is complementary to other resources that provide access to data, including the one we developed at ICR called ProbeMiner (https://probeminer.icr.ac.uk) which is a community resource providing evaluation of chemical probes based on multi-parameter statistical analysis of large-scale, publicly available medicinal chemistry literature plus additional journals with relevant data^13^.

As well as supporting efforts to increase the ‘probed proteome’ by working closely with Target 2035, a major objective now is to make the broader biomedical community more aware that the Portal exists and how we can help. The Portal is now supported by a Wellcome Biomedical Resource Award, which includes funding for outreach activities. We tweet (@Chemical_probes) and publish news pieces and blogs. We run webinars, often in partnership with Target 2035. We talk at conferences and publish articles. And our intended audience is not just the biologists and other active researchers. It’s also the journal editors, the funders, the regulators, the whole science community. We find that for some of these audiences at-a-glance checklists of chemical probe properties are useful.

**—** The Target 2035 initiative arose from discussions among a group of scientists who met in 2018 to discuss the need and feasibility of developing a chemical probe for each protein encoded by the human genome^14, 15^. It’s really just a very small portion of the proteome that we are able to target pharmacologically right now. If you look at the OMIM [https://www.omim.org/] (Online Mendelian Inheritance in Man) database, almost every human gene/protein has some kind of a link to disease. There’s a treasure trove in our proteome of drug targets that are not the typical classes. But we don’t have the chemical probes to determine the functions of these proteins, or to evaluate their roles in a disease model or disease cell type.

Target 2035 is meant to galvanize the global research community to think about the potential of chemical probes and to spur collaboration in the relevant subareas: biochemistry, structural biology, medicinal chemistry, enzymology, pharmacology, to create these ‘missing’ chemical probes. Bringing researchers together is very important. Particularly biochemists and cell biologists with medicinal chemists, who typically don’t work in the same academic lab. Each area involves very different training, but we need these groups to work together. In the future, the characterization of chemical probes will also increasingly involve methodologies like proteomics, and computational methods are going to be key in the coming years as AI and machine learning methods continue to improve. We need the labs with the relevant expertise to be involved in the chemical probes community as well.

The goal of Target 2035 for the first five years, until 2025, is really just to make the scientific community—including researchers, but also funding agencies, regulators and publishers—aware of the problem and of the potential. The SGC is organizing this initiative, but it must be a truly global effort. We want to help facilitate interactions amongst groups with these various types of expertise, and to encourage best practices as we just discussed. Hopefully, we will make the community and the research funders recognize the potential and generate enough evidence so that by 2025 the funders around the world will start to support coordinated projects in this area.

As already mentioned, one of the tools that we use are webinars. These are recorded and anyone can access them. The audience for these seminars comprises mostly the people working in the field of chemical probes and drug discovery. Presentations and panel discussions include chemistry strategies, enzymology and inhibition of specific target classes, computational methods, etc. A lot of the concepts that we mentioned today are discussed in detail. It’s a good overview for somebody who does want to work in this field or is working in one part of the field but wants to learn about others: for example you’re a biochemist, but you want to learn about some of these chemistry concepts and important aspects, or vice versa.

7. What other advice do you have for those who are only starting or want to start working with chemical probes?

— Clearly there’s an awful lot of factors to consider in evaluating and selecting a chemical probe for use in vitro and in animals—and this can be challenging, especially for the non-expert. We are conscious of the danger of appearing to sound overly preachy or evangelical. Experts dictating how things need to be done—and perhaps inadvertently making it too difficult for researchers to publish a new chemical probe or to make a choice from a range of probe options. That’s not the intention. But we’ve certainly had researchers and journal editors tell us that very rigorous criteria can make it difficult for small labs, especially perhaps early career scientists, to meet the exacting standards and to publish their initial work involving new chemical probes. So it’s important to apply a common sense, fit-for-purpose approach^5^. Best practice guidelines are valuable but they are guidelines not absolute rules. Especially in the early days of probe evolution against a particular target, there’s no doubt that suboptimal reagents can be valuable as ‘pathfinder compounds’ en route to more stringently qualified chemical probes that emerge later. These pathfinders may not be as potent as you would like, and they may have some off-target effects, or may not be suitable for animal work and so on, but they’re still useful. As long as we understand that they’re not perfect, that the properties and limitations are made clear, and that the publication does not over claim, there is certainly a place for these. Sometimes you can’t always get to a perfect chemical probe with all the essential criteria met.

— Also to mention here is that while individual small labs can absolutely make important contributions, discovering and evaluating high-quality chemical probes can proceed very quickly and efficiently through multi-disciplinary collaborations and interactions between chemists, biologists, pharmacologists, structural biologists, in vivo modellers and disease experts and so on. It really is team science, so even if you don’t have a particular expertise in your own lab or your own institution, you probably can find a way to collaborate with other researchers, and get help with chemical probe development and characterization. Smaller labs and early career researchers can play valuable roles and will find that this is exciting and rewarding science in which contributions are appropriately acknowledged.

*This interview was conducted by Katarzyna Marcinkiewicz*.
